# Key Biomarker Correlations in Cutaneous Melanoma: Implications for Diagnostic, Prognostic, and Therapeutic Strategies—A Retrospective Single-Centered Study

**DOI:** 10.3390/medicina61101733

**Published:** 2025-09-24

**Authors:** Mariana Costache, Ancuța-Augustina Gheorghişan-Gălățeanu, Diana Derewicz, Cătălin Cîrstoiu, Andreea Ilieșiu

**Affiliations:** 1 Department of Pathology, Carol Davila University of Medicine and Pharmacy, 020021 Bucharest, Romania; 2 Department of Pathology, University Emergency Hospital, 050098 Bucharest, Romania; 3 Department of Cellular and Molecular Biology and Histology (Ret.), Carol Davila University of Medicine and Pharmacy, 020021 Bucharest, Romania; 4 Parhon National Institute of Endocrinology (Ret.), 011863 Bucharest, Romania; 5 Department of Pediatrics, Carol Davila University of Medicine and Pharmacy, 020021 Bucharest, Romania; 6 Department of Pediatric Hematology and Oncology, Marie Sklodowska Curie Clinical Emergency Hospital, 041447 Bucharest, Romania; 7 Department of Orthopedics and Traumatology, “Carol Davila” University of Medicine and Pharmacy, 030167 Bucharest, Romania; 8 Department of Orthopedics and Traumatology, University Emergency Hospital, 050098 Bucharest, Romania

**Keywords:** cutaneous melanoma, biomarkers, immunohistochemistry, prognosis

## Abstract

*Background and Objectives*: Cutaneous melanomas are highly aggressive and prevalent malignancies that often require complex diagnostic and therapeutic procedures. Mounting evidence supports the utility of several biomarkers for improving diagnostic accuracy and treatment decisions. In this study, we aimed to evaluate the correlations between various histopathological and immunohistochemical markers to better understand melanoma development and its subsequent behavior. *Materials and Methods*: We conducted a retrospective study on 59 patients diagnosed with cutaneous melanoma to establish the significant correlations between clinical, histopathological, and immunohistochemical markers. *Results*: The mean age of the patients was 60.15 years, with a male-to-female ratio of 1.27:1. Our results demonstrate significant correlations between proliferative activity, evaluated both as mitotic counts and Ki-67 index, and clinicopathological parameters. Other significant correlations between melanoma immunohistochemical markers such as Melan-A, HMB45, S100 or PRAME and Breslow depth highlight their potential not only for diagnostic but also for prognostic purposes. Additionally, the significant negative correlations between p16 and patient age, Breslow depth, and the Ki-67 index emphasize the predictive value of this still insufficiently described parameter. *Conclusions*: Taken together, these observations underscore the importance of integrating biomarker evaluation into melanoma management, enabling more precise prognostication and the development of individualized treatment strategies.

## 1. Introduction

Cutaneous melanoma is a malignant tumour that originates in melanocytes, the cells in the basal layer of the epidermis responsible for producing melanin [1,2]. Melanoma ranks as the third most frequently diagnosed type of skin cancer, after basal cell carcinoma and squamous cell carcinoma [3]. Most deaths from skin cancer are caused by cutaneous melanomas, with the 5-year survival rate for stage IV tumours being as low as 23% [4]. Melanoma subtypes that occur outside the skin, such as mucosal and ocular melanomas, are generally associated with a poorer prognosis [5]. In the United States alone, an estimated 106,110 new cases of invasive melanoma and 7180 related deaths were expected in 2021. Globally, GLOBOCAN 2020 data reported 324,635 melanoma cases, making up 1.7% of all cancer diagnoses, and 57,043 deaths, corresponding to 0.6% of worldwide cancer mortality [6]. These statistics highlight the significant impact of melanoma on global health and emphasize the need for ongoing research and improved treatment strategies.

Melanoma is distinct from other skin cancer types due to its significant ability to metastasize not only locally but also to regional and distant areas. The likelihood of metastasis is closely associated with the depth of tumour invasion into the skin and the presence of ulceration in the primary lesion. Early metastatic progression involves several biological steps, including tissue invasion, the formation of new blood vessels (angiogenesis), entry into the bloodstream or lymphatics (extravasation), dissemination, and eventual colonization of distant organs [7]. It is important to note that patients who initially show no signs of lymph node involvement—whether clinically or through negative sentinel lymph node biopsies—can still develop distant metastases. This highlights the aggressive nature of melanoma and underscores the critical importance of early detection and continuous ongoing monitoring [8]. Among noncontiguous sites, lymph nodes are the most common targets for melanoma spread. Sentinel lymph nodes (SLNs) are particularly important because they are usually the first nodes to drain lymphatic fluid from the tumour site. They serve as key indicators of the potential for metastasis [9,10].

Cutaneous melanoma is diagnosed through a skin biopsy, which must assess the depth of tumour invasion, known as Breslow thickness. This measurement is the most significant prognostic factor for the disease. In addition to Breslow thickness, other important indicators such as the mitotic rate (number of mitoses per mm^2^) and the presence or absence of ulceration, play a crucial role in determining appropriate treatment strategies [11,12].

A range of immunohistochemical markers can support both the diagnosis and prognosis of cutaneous melanoma. Among the most widely utilized diagnostic markers are Melan-A, HMB45, and S100, which generally offer high specificity and sensitivity. However, their reliability may diminish in cases of undifferentiated or dedifferentiated tumours [13]. This has led to ongoing investigations into whether the loss of these markers holds prognostic value. For example, the prognostic relevance of Melan-A staining intensity remains uncertain and is still under discussion [14]. To address these limitations, additional markers have been introduced. One such marker is SOX10, a transcription factor belonging to the SOX gene family, characterized by a 79-amino-acid DNA-binding domain similar to the high-mobility group (HMG) box found in the sex-determining region Y (SRY) gene—hence the name SOX, derived from ‘Sry bOX’. These transcription factors play a crucial role in regulating cellular processes, including stemness, fate determination, and differentiation. The growing number of developmental disorders linked to mutations in SOX genes reflects their fundamental biological roles [15,16,17,18]. SOX10 is particularly important in melanoma due to its regulatory influence on the MITF pathway, with evidence of direct cross-activation between SOX10 and MITF. This interaction suggests that preserving wild-type SOX10 function may support the survival and proliferation of melanoma cells. Nevertheless, certain mutations in SOX10 may exaggerate this pathway’s activity, thereby promoting melanomagenesis in specific scenarios. A study by Cronin et al. identified SOX10 mutations in 6 of 55 primary melanoma samples and in 3 of 50 metastatic cases [19,20]. As a result, SOX10 has become a key immunohistochemical marker in the diagnosis of cutaneous melanoma. Additionally, research by Gambichler et al. indicated that SOX10 expression is linked to metastatic progression [21].

PRAME (Preferentially Expressed Antigen in Melanoma) is among the more recently adopted immunohistochemical markers for cutaneous melanoma. It was initially discovered through the study of tumour-reactive T-cell clones derived from a patient with metastatic melanoma. Although originally associated with cutaneous melanoma, PRAME is also found in ocular melanoma and a range of other non-melanocytic malignancies, such as non-small cell lung cancer, breast and renal carcinomas, ovarian cancer, leukemia, synovial sarcoma, and myxoid liposarcoma [22,23,24]. In contrast, its expression in normal tissues is highly restricted—limited primarily to the testis, ovary, placenta, adrenal glands, and endometrium [25]. Due to its selective expression, PRAME is classified as a cancer-testis antigen (CTA), making it an attractive target for immunotherapeutic strategies. Current clinical trials are investigating the potential of Cancer Testis Antigens (CTAs), including PRAME, in cancer treatment. Beyond its therapeutic relevance in metastatic melanoma, PRAME is also a valuable prognostic marker—especially in class 1 uveal melanoma, where it helps estimate the risk of metastasis [24,26]. Furthermore, PRAME is included in a 12-gene expression panel used for prognosis in uveal melanoma and in a 23-gene diagnostic panel for cutaneous melanoma. It is also one of the two genes featured in a non-invasive molecular assay developed to guide clinicians in deciding whether to biopsy suspicious melanocytic lesions [27,28].

Ki-67 was first identified as a nuclear antigen present in Hodgkin lymphoma cells [29], and is known for its elevated expression in proliferating cells, while being markedly reduced or absent in quiescent cells in the G0 phase of the cell cycle [30]. Due to this pattern, Ki-67 has become a widely accepted marker for evaluating cell proliferation and is commonly used in the histological grading of various malignancies, with numerous studies confirming its prognostic relevance [31,32,33]. While Ki-67 has a well-established role in other cancers, its prognostic value in cutaneous melanoma remains unclear due to inconsistent findings in the literature. Nevertheless, more recent investigations suggest that Ki-67 may be helpful for risk stratification in melanoma patients [34,35,36].

Another crucial factor in melanoma progression is the mutation of the CDKN2A gene, which encodes the tumour suppressor protein p16 [37]. Loss of p16 expression is frequently observed in cutaneous melanomas and is considered a distinguishing feature when compared to benign nevi [38]. Reduced levels of p16 have also been linked to poorer prognosis in advanced melanoma cases [39]. However, a 2018 meta-analysis concluded that p16 alone may not be a reliable marker for differentiating between melanomas and nevi [40]. Intriguingly, newer studies have shown that p16 can, in some cases, be paradoxically overexpressed in cutaneous melanomas, suggesting a more complex role in tumour biology [41].

This article examines the relationships between different histopathological and immunohistochemical characteristics in cutaneous melanomas. The aim is to enhance our understanding of how these parameters interact to promote the disease’s aggressiveness. Furthermore, this knowledge can be utilized for prognostic and therapeutic purposes.

## 2. Materials and Methods

This retrospective study included 59 patients diagnosed with primary cutaneous melanomas at the University Emergency Hospital of Bucharest, Romania, between January 2012 and December 2023. The research adheres to the main principles outlined in the Declaration of Helsinki and received approval from the Ethics Committee of the University Emergency Hospital of Bucharest, Romania. Clinical data were extracted from relevant files. All data processing complied with current GDPR, and informed consent was obtained from all patients included in the study.

The inclusion criteria were as follows:

- Only primary tumours that were completely surgically removed.
- No prior therapy had been administered.
- Available follow-up information, including full-body CT scans after surgery to detect metastatic lesions and survival data in 2025;
- Availability of immunohistochemical analyses for standard markers, including Ki-67, along with sufficient tissue samples for additional studies on PRAME and p16.

Initially, 136 cases were screened, but after applying the inclusion criteria, only 59 cases remained eligible for analysis.

The resection specimens were processed according to standard histopathology protocols. For each patient, we collected the following variables: age, gender, primary tumour location, histological subtype of the melanoma, and Breslow depth. All cases were reviewed before conducting statistical analyses, and melanoma subtypes were classified according to the fifth edition of the WHO Classification of Tumours Editorial Board—skin tumours [42]. TNM staging was assessed using the eighth edition of the American Joint Committee on Cancer (AJCC) cancer staging manual [43]. Immunohistochemical analyses were performed for the following markers: HMB45 (mouse monoclonal antibody, Biocare Medical, Pacheco, California, clone HMB45), Melan = A (mouse monoclonal antibody, Biocare, cone A103), PRAME (rabbit monoclonal antibody, Biocare, clone EPR20330), SOX10 (mouse monoclonal antibody, Biocare, clone BC34), S100 (mouse monoclonal antibody, Biocare, clone 4C4.9), p16 (mouse monoclonal antibody, Biocare, clone BC42) and Ki-67 (rabbit monoclonal antibody, Biocare, clone SP6). Two pathologists (M.C. and A.I.) independently analyzed all immunohistochemical markers, and any discrepancies in their results were resolved through a joint review of the slides. All immunohistochemical markers included in this analysis were reported as continuous variables. The Ki-67 index value was determined using the hotspot method. The evaluation of all melanocytic markers and p16 was expressed as a percentage of positive tumour cells, ranging from 0% (completely negative) to 100% (strong and diffuse positivity throughout the entire section).

Descriptive statistics were performed for continuous variables. Continuous variables were assessed for normality using the Shapiro–Wilk test. Because the mitotic counts, Breslow depth, Ki-67 index, and expression of Melan-A, HMB45, S100, PRAME, and p16 were not normally distributed, the Correlation Matrix was determined using Spearman correlation, providing ρ values and *p*-values, with significance considered at *p* < 0.05. Logistic regression was performed to identify significant prognostic factors for survival, yielding odds ratios (OR), 95% confidence intervals (95% CI), and *p*-values. Statistical analysis and figure plotting were performed using GraphPad Prism 10.5.0 (GraphPad Software, Inc., San Diego, CA, USA).

## 3. Results

### 3.1. Demographic and Clinical Characteristics of the Study Population

**Figure 1:**
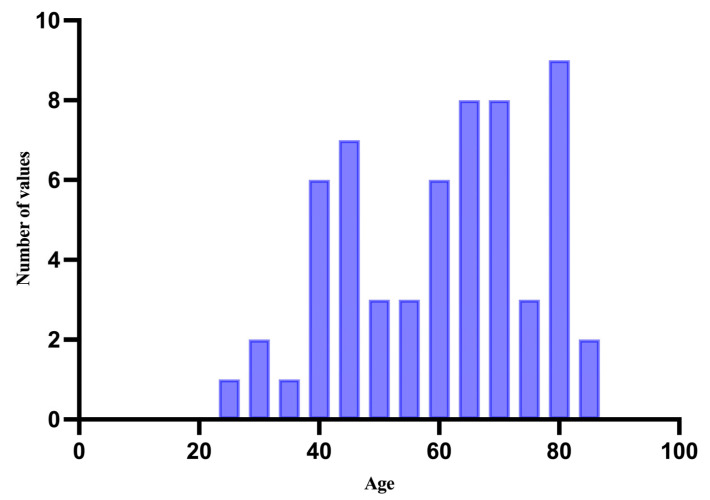
Distribution of cases by age.

The mean age of the group was 60.15 years, with the youngest patient being 24 years old and the oldest 86 years old, with a standard deviation of 15.85 (Figure 1).

The study included 33 females and 26 males, resulting in a male-to-female ratio of 1.27:1. The mean age for females was 59.61 years, and for males it was 60.85 years.

**Figure 2:**
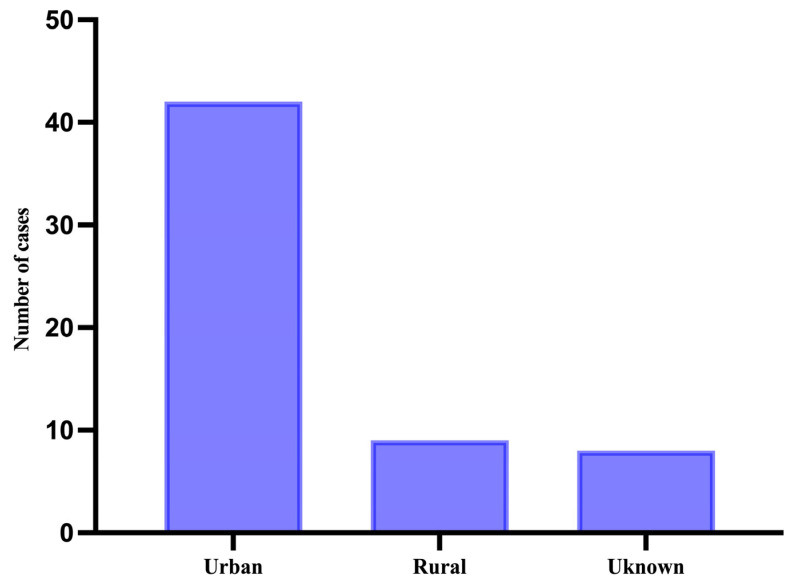
Distribution of cases based on demographic region.

In terms of demographics, 9 patients were from rural areas (15.3% of all patients), 42 were from an urban setting (71.2% of all patients), and 8 did not disclose their environment (13.6% of all patients) (Figure 2).

**Figure 3:**
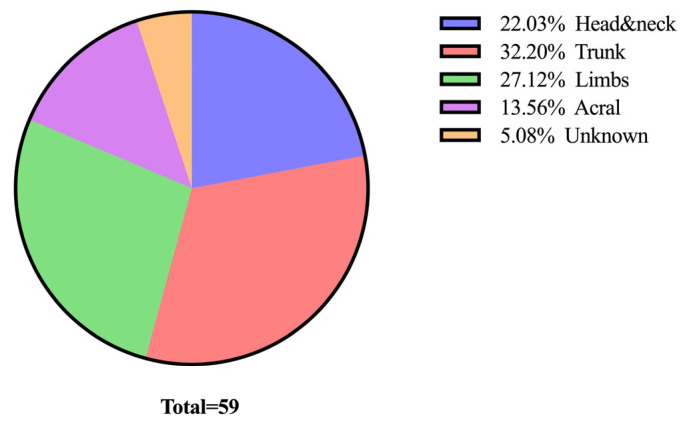
Distribution of cases based on tumour location.

The most common location for tumours was the trunk, which accounted for 19 cases. This was followed by the limbs with 16 cases, and the head and neck region, which had 13 cases. Tumours were also frequently located in the acral region, with 8 cases reported. Finally, in 3 cases, the tumour location was not specified (Figure 3).

**Figure 4:**
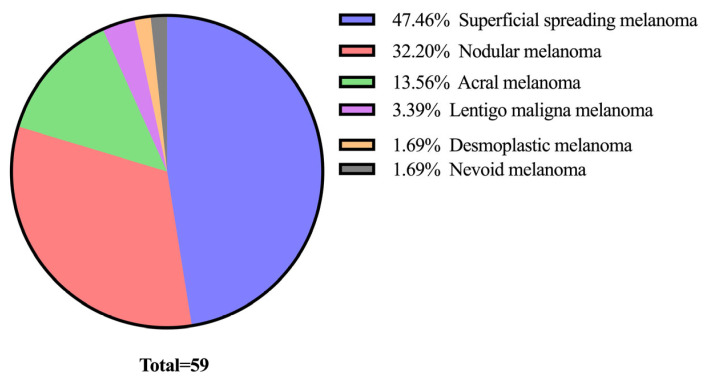
Distribution of cases based on histological subtypes of cutaneous melanomas.

We analyzed the distribution of melanoma cases based on their histological subtypes. Superficial spreading melanoma was the most common subtype, with 28 cases. In contrast, the least common subtypes were desmoplastic melanoma and nevoid melanoma, each representing only 1 case (Figure 4).

**Figure 5:**
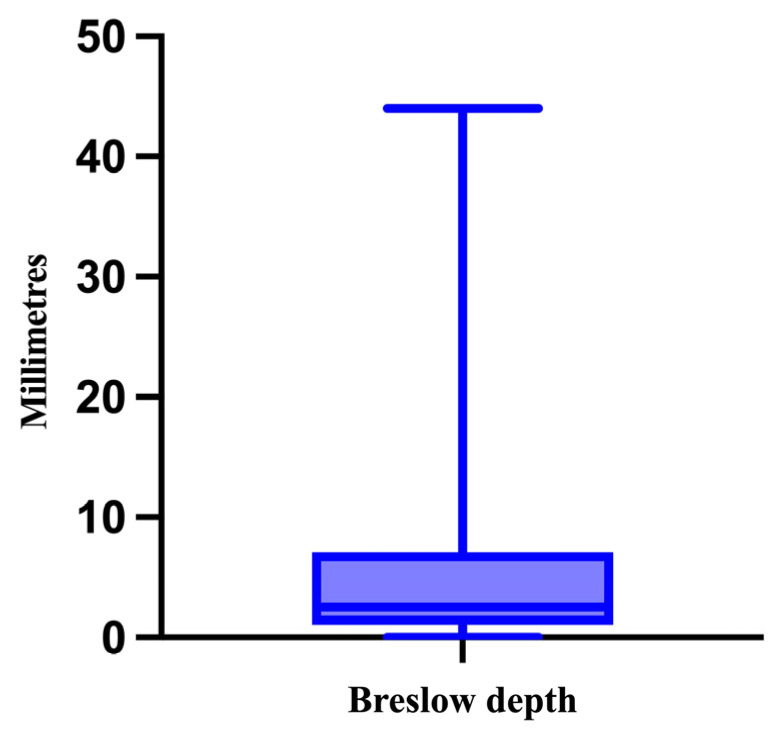
The median, minimum, and maximum values of the Breslow depth.

The next histopathological feature that was analyzed was the Breslow depth of invasion, ranging from 0 mm for in situ melanomas to 44 mm, with a median of 2.5 mm (Figure 5).

**Figure 6:**
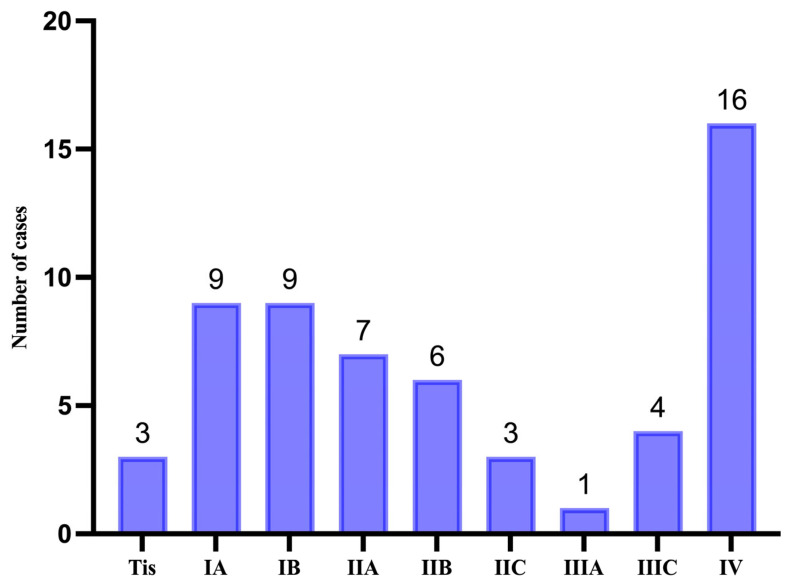
Distribution of cases based on TNM staging.

Based on the Breslow depth of invasion and clinical assessment, the TNM staging was established. Out of the cases reviewed, 16 (n = 16) were diagnosed with metastatic disease, as illustrated in Figure 6.

As of June 2025, 30,51% of the patients (n = 18) died due to disease progression.

**Figure 7:**
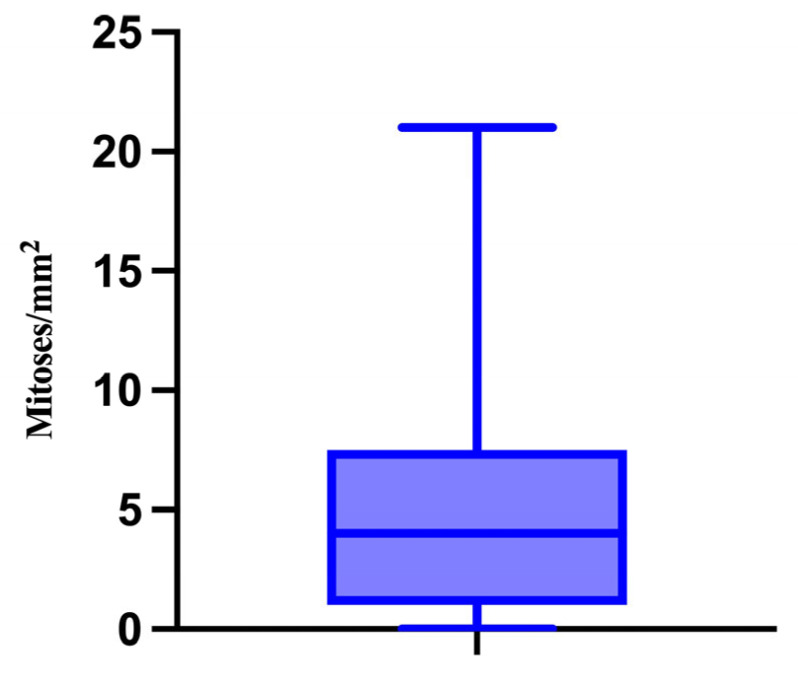
The median, minimum, and maximum values of the mitotic index.

Furthermore, we also analyzed the number of mitoses/mm^2^. The minimum value was 0 and the maximum value was 21, with a median of 4 mitoses/mm^2^ (Figure 7).

**Figure 8:**
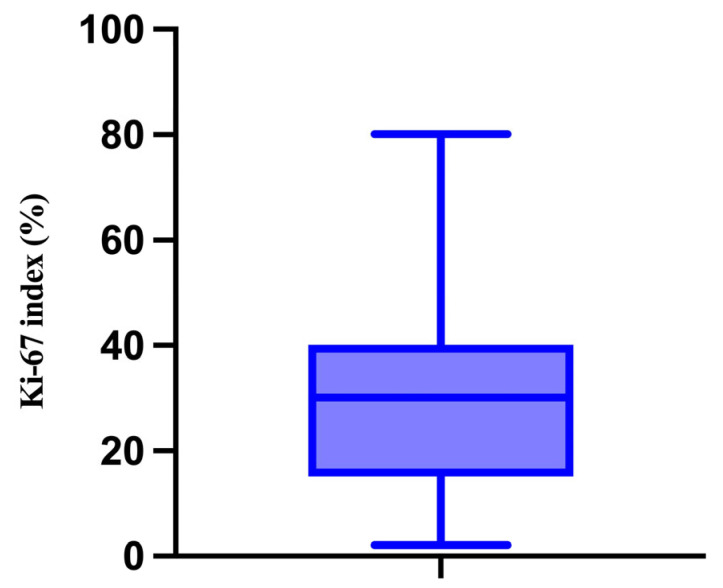
The median, minimum, and maximum values of the Ki-67 index.

We also evaluated the proliferative activity of the melanomas by performing immunohistochemical analysis for Ki-67. In this context, the minimum value was 2% and the maximum value was 80%, with a median of 30% (Figure 8).

**Figure 9:**
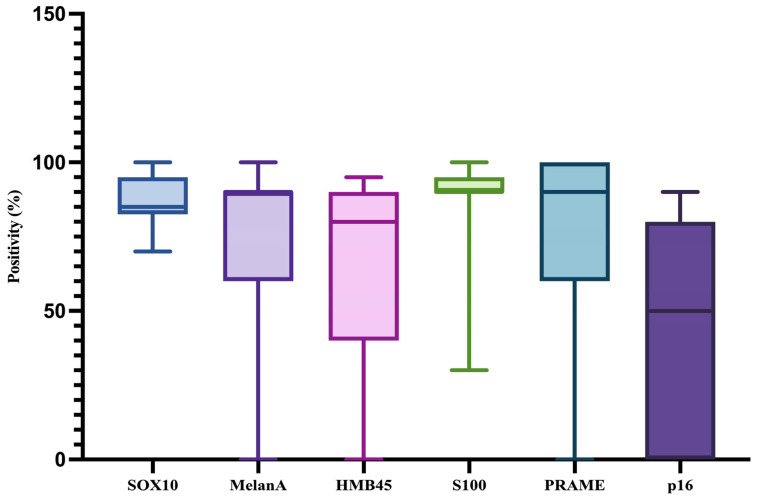
Graphical representation of the minimum, maximum, and median percentage of positive cells for SOX10, Melan-A, HMB45, S100, PRAME, and p16.

In addition, we have analyzed the immunohistochemical expression of the following markers: SOX10, Melan-A, HMB45, S100, PRAME, and p16. The minimum, maximum, and median percentage of positive cells for each of them are presented in Table 1 and in Figure 9.

### 3.2. Description and Analysis of the Correlation Matrix

**Figure 10:**
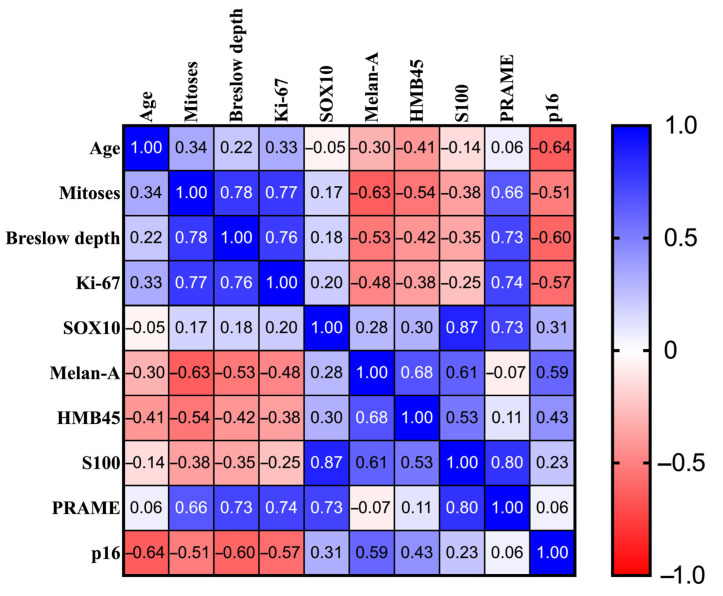
The Spearman Correlation Matrix coefficient values.

We have developed a Correlation Matrix using our patient database, which illustrates the strength and direction of relationships between various clinical and histopathological parameters. Each cell in the matrix displays the correlation coefficient between two parameters, with the values ranging from −1 to 1. The color scale indicates both the strength and direction of the correlation: blue hues represent positive correlations, while red hues indicate negative correlations. The intensity of the color reflects the magnitude of the correlation (Figure 10).

The Correlation Matrix provides a comprehensive overview of the relationships among various tumour parameters. Key findings from the matrix include several high positive correlations. Firstly, the mitotic count is strongly correlated with Ki-67 (ρ = 0.77) and also shows a strong correlation with the Breslow depth of invasion (ρ = 0.78) and PRAME expression (ρ = 0.66). Notably, the number of mitoses has a negative correlation with the expression of Melan-A (ρ = −0.63), HMB45 (ρ = −0.54), S100 (ρ = −0.38), and p16 (ρ = −0.51). Additionally, the Breslow depth of invasion is strongly correlated with the Ki-67 index (ρ = 0.77) and PRAME expression (ρ = 0.66). It is negatively correlated with the expression of Melan-A (ρ = −0.53), HMB45 (ρ = −0.42), S100 (ρ = −0.35), and p16 (ρ = −0.51) as well. Similarly, the Ki-67 index correlates positively with PRAME (ρ = 0.72) while demonstrating negative correlations with the expression of Melan-A (ρ = −0.56), HMB45 (ρ = −0.46), S100 (ρ = −0.26), and p16 (ρ = −0.47). The expression of p16 shows a moderate positive correlation with the expression of Melan-A (ρ = 0.59) and HMB45 (ρ = 0.43). It is weakly correlated with the expression of SOX10 (ρ = 0.31) and S100 (ρ = 0.23). Interestingly, there were strong negative correlations between p16 expression and both the age of the patients (ρ = −0.65) and Breslow depth (ρ = −0.60). Additionally, p16 expression is moderately negatively correlated with the number of mitoses (ρ = −0.51) and the Ki-67 index (ρ = −0.57).

In examining the correlations among various melanocytic markers, the weakest correlations were observed between HMB45 and PRAME (ρ = 0.11) and between HMB45 and SOX10 (ρ = 0.30). In contrast, Melan-A, HMB45, and S100 exhibited strong positive correlations, indicating that an increase in the intensity of one marker is associated with an increase in the intensity of the others. However, Melan-A has only a weak correlation with SOX10 (ρ = 0.28) and displays a very weak negative correlation with PRAME (ρ = −0.07). Furthermore, the expressions of S100, SOX10, and PRAME also demonstrate strong positive correlations with each other. Gaining insights into these correlations can provide valuable information about tumour behavior and assist in clinical decision-making. In this context, the *p*-values of the Spearman correlations are presented in Table 2.

As illustrated in Table 2, several statistically significant correlations are evident. Firstly, the number of mitoses shows a significantly positive association with patient age, Breslow depth of invasion, Ki-67 index, and PRAME expression. On the other hand, there are significant negative correlations between the mitotic index and the expression of Melan-A, HMB45, and S100. Furthermore, an increase in Breslow depth is significantly correlated with both an increase in the Ki-67 index and PRAME expression, as well as a decrease in the expressions of Melan-A, HMB45, S100, and p16. There are also significant negative correlations between the Ki-67 index and the expressions of Melan-A, HMB45, and p16. Notably, positive correlations between the Ki-67 index and both patient age and PRAME expression are also significant. Regarding melanocytic markers, SOX10 is positively correlated with PRAME, while Melan-A expression is significantly correlated with both HMB45 and S100. Additionally, HMB45 is also correlated with S100. Finally, a significant negative correlation exists between p16 expression and patient age.

The final section of this research analyzed how the parameters in the correlation matrix affect patient outcomes through univariate logistic regression (Table 3).

As shown in Table 3, neither the patients’ age nor the expression levels of SOX10 and S100 significantly predict survival. In contrast, Breslow depth, mitotic count, Ki-67 index, and elevated PRAME expression are identified as significant negative prognostic factors. Conversely, higher expression levels of Melan-A, HMB45, and p16 are considered significant positive prognostic factors in univariate analysis.

## 4. Discussion

The present study focuses primarily on immunohistochemical markers from a pathologist’s perspective, particularly on the interactions between these markers and their potential roles in influencing prognosis, staging, and even treatment decisions in cutaneous melanoma.

At the beginning of this study, the demographic and clinical characteristics of the patients were examined. The average age of the patients in this study is 60.15 years, with male patients being slightly older on average, a finding that aligns with numerous other studies [44,45,46]. While the global incidence of melanoma is rising among women, it still predominantly affects men [47,48], a trend also observed in the present study. The most common sites for these tumours were the trunk and the limbs. Although reports may vary, these locations are typically the two most frequent sites for cutaneous melanomas. In general, the trunk is the most common site for male patients, while the lower limbs are more frequently affected in female patients diagnosed with cutaneous melanomas [49,50,51].

Superficial spreading melanoma was the most common subtype, which is consistent with findings from other studies [51,52]. However, the low incidence of lentigo maligna melanoma and the high incidence of acral lentiginous melanoma, in contrast to other studies [52,53], are observations that warrant further investigation.

The correlation network graph and another. The correlation matrix provides valuable insights into the interactions between various clinical and histopathological parameters, highlighting potential areas for further research and clinical evaluation. High correlation values indicate strong relationships, which may point to underlying biological connections or shared pathways that influence these factors. By understanding these correlations, we can better characterize tumours, assess prognosis, and develop treatment plans, ultimately leading to improved patient outcomes. The importance of thoroughly assessing these parameters is also highlighted by the significant associations with patient survival, as revealed through logistic regression analyses.

Currently, gene expression profiling is one of the most widely used tools for characterizing malignant tumours, including melanoma. These assays aim to provide prognostic value—such as the DecisionDx-Melanoma test (31-GEP, Castle Biosciences, Friendswood, Texas, USA)—which may help guide clinical decision-making. However, the National Comprehensive Cancer Network (NCCN) [54], the American Academy of Dermatology (AAD) [55,56], and the most recent consensus from the Society of Surgical Oncology (SSO) [57] state that while these types of assays hold significant potential, they are currently limited to research use only in cutaneous melanoma. To date, it has not been demonstrated that they can reliably predict sentinel lymph node status, improve current risk stratification or follow-up protocols, or influence therapeutic decisions.

In all current classification systems, there are no immunohistochemical markers officially used for prognostic purposes. Our study focuses on these markers and explores in depth the correlations between them.

A recent study evaluated the role of PRAME and Ki-67 in predicting sentinel lymph node outcomes, highlighting the potential importance of these immunomarkers in staging and, consequently, in treatment decisions and patient outcomes [36]. The associations between well-established prognostic factors, such as Breslow thickness, and markers like PRAME or p16, may play a significant role in staging and could further guide treatment strategies for patients. The clinical guidelines for diagnosing, staging, and treating melanoma are internationally standardized to ensure greater consistency in the management of this oncological disease. However, research in this field is rapidly evolving, and markers such as PRAME have emerged as promising candidates for diagnostic, prognostic, and even therapeutic use in cutaneous melanoma [58]. Studies such as that by Wermke et al. [59] have shown that PRAME-positive tumours, including melanoma, exhibit an overall response rate of 52.5% to PRAME-targeted T-cell immunotherapy. Another promising therapeutic avenue involving PRAME is the development of mRNA vaccines. Weber et al. [60] reported encouraging results—now advancing to phase III trials—for the mRNA-4157/V940 vaccine, a personalized treatment that targets multiple antigens (including PRAME) and is administered in combination with pembrolizumab.

In our study, PRAME expression is significantly correlated with the mitotic count, and the Ki-67 index, and similar observations have been reported by other researchers [36,61]. This suggests that more aggressive melanomas may exhibit higher levels of PRAME expression. Our study is among the first to identify a significant correlation between PRAME expression and the Breslow depth of invasion, as previous research has not demonstrated such significance [61,62]. These findings are particularly significant because the prognostic value of this marker in cutaneous melanomas is still debated. While PRAME expression has been linked to a negative prognostic in uveal and mucosal melanomas, this association has not been established for cutaneous melanomas [63]. Nevertheless, based on logistic regression analysis, the increase in PRAME expression is significantly associated with decreased survival in our patients. Therefore, further studies are necessary to comprehensively address this issue. The significant positive correlation between PRAME and SOX10 is also clinically relevant. This strong relationship indicates a possible interaction in the development of melanoma. Since both markers are involved in melanoma characterization and prognosis, their correlation may enhance the accuracy of diagnostic and prognostic assessments.

A significant finding is the strong correlation between Breslow depth, and both the Ki-67 index and the mitotic rate. This correlation is supported by various studies [35,64,65,66]. Both markers are crucial for assessing tumour aggressiveness, as also highlighted in this paper by their significant negative associations with patient outcome. Elevated levels of Ki-67 and higher mitotic rates are typically associated with more aggressive tumours, which may necessitate more intensive treatment strategies. These findings support the use of these markers in routine clinical practice to guide therapeutic decisions and monitor treatment responses. However, several studies have shown that, unlike the mitotic count, the Ki-67 index is not an independent prognostic factor in cutaneous melanoma [64,67,68,69,70]. In this context, performing additional analysis of Ki-67 for prognostic purposes might not be necessary. Nevertheless, evaluating Ki-67 can provide valuable insights for therapeutic strategies. Since Ki-67 is highly expressed in proliferating cells and downregulated in resting cells, it serves as a target for treatments aimed at controlling cell growth. Drugs that modulate Ki-67 activity have the potential to inhibit cancer cell proliferation, offering a targeted approach to cancer therapy [71]. The mitotic count and the Ki-67 index are positively correlated with patient age. Several authors have reported increased proliferative activity in cutaneous melanomas among older individuals [72,73]. However, the exact significance of this association remains unclear. Studies analyzing the prognostic value of age in patients with cutaneous melanoma have shown conflicting results [73,74,75]. In this study, the age of the patients was not found to be significantly associated with overall survival.

The loss of p16 expression has been linked to poor outcomes in cutaneous melanomas, as well as an increase in Breslow thickness [39,76]. In this study, statistically significant negative correlations were found between p16 and both Breslow depth and the Ki-67 index. Furthermore, increased p16 expression was a significant positive prognostic factor in our analysis. These results highlight the need for further research into the prognostic implications of p16 in cutaneous melanoma. Obadofin et al. reported finding no significant association between p16 expression and Breslow depth [77], while Fauri et al. argue that p16 expression is not an important prognostic factor in cutaneous melanomas [78]. In contrast, Gkionis et al. demonstrated that more invasive melanomas tend to lose p16 expression [39]. Additionally, Ghiorzo et al. found a statistically significant correlation indicating that the loss of p16 expression is a negative prognostic factor [79]. The relationships between p16 and the Ki-67 index, as well as mitotic counts, have been rarely evaluated; however, existing data confirm the significant negative correlation found in this study [80,81]. To the best of our knowledge, this is the first study to demonstrate a strong and significant negative correlation between p16 expression and patient age. Although previous research has not found a correlation between these two parameters, Obadofin et al. observed a trend indicating that p16 expression decreases with increasing patient age, though this difference was not statistically significant [77,78,82,83]. p16 may also be relevant to therapeutic approaches, such as the use of CDK4/6 inhibitors [84] or p16-related peptides [85]; however, studies are still ongoing. As a consequence, further research is warranted to explore this potential correlation and its implications for therapy.

In our study of melanocytic markers, we confirmed strong and statistically significant correlations between the intensity of HMB45, Melan-A, and S100. Notably, both HMB45 and Melan-A showed significant negative correlations with the Breslow depth, mitotic rate, and Ki-67 index. Additionally, we found a statistically significant negative correlation between S100 expression and Breslow depth. These findings suggest that more advanced tumours tend to lose expression of conventional melanocytic markers. This observation is also supported by the significant positive association between the expression of HMB45 and Melan-A, as well as overall survival. Understanding these relationships can enhance our knowledge of tumour progression, as the phenomenon of undifferentiation in cutaneous melanomas is believed to contribute to resistance to therapy. Furthermore, undifferentiated and dedifferentiated melanomas often occur in older individuals [13], which may explain the significant negative correlation observed between HMB45 and Melan-A and patient age.

Along with the immunohistochemical marker studied, we also mention PD-L1. Stage IV cutaneous melanoma is eligible for immunotherapy with agents such as nivolumab, pembrolizumab, and atezolizumab, which target the PD-1/PD-L1 pathway [86]. Recent studies have highlighted a correlation between PD-L1 expression and a favorable response to targeted immunotherapy [87], underscoring the crucial role that immunohistochemical markers can play in guiding therapeutic decisions.

Apart from immunohistochemical markers, new serum biomarkers are emerging in other oncological diseases—for example, butyrylcholinesterase, which shows great potential in predicting complications and poor outcomes. Being a serum enzyme, it can be easily assessed by clinicians, and low serum levels have been associated with poor prognosis in colorectal cancer [88]. This enzyme has also been studied in melanoma patients, where significant correlations were found between disease progression (including metastasis) and serum butyrylcholinesterase levels [89]. More recent studies have focused on fluorescence-based strategies for the early detection of hepatic melanoma metastases. These promising in vivo results highlight the importance of ongoing research into new biomarkers with clinical relevance [90].

This study has several limitations due to its retrospective design that must be acknowledged. First, the analysis is based on a relatively small number of cases from a single center. Second, selection bias may exist, as only patients who underwent complete surgical excision and had adequate tissue or records were included. Third, while key clinicopathologic variables were mostly complete, the potential for unrecognized missing data or case attrition cannot be ruled out. Finally, because this was not a prospective or randomized study, causal relationships between variables cannot be definitively established. Accordingly, any prognostic associations observed should be considered hypothesis-generating. To validate our findings and assess their clinical significance, future prospective studies with larger cohorts and standardized data collection are needed. One way to strengthen the reliability of our conclusions is to validate our results with a larger, independent dataset, such as the TCGA melanoma cohort. However, it is important to note that our study focuses exclusively on primary cutaneous melanomas, whereas the TCGA cohort includes about 20% primary tumours and 80% metastases [91]. Another strategy to address this limitation would be to increase the number of enrolled patients through a national, multicenter study, which could further enhance the robustness of our findings.

After careful consideration, our findings have significant clinical implications. The relationships between various markers underscore the importance of a multifaceted approach to melanoma diagnosis and treatment. By integrating these markers into routine clinical practice, clinicians can gain a more comprehensive understanding of tumour biology, leading to more effective patient management and improved prognostic accuracy. Moreover, the strong correlations identified in our study suggest potential targets for new therapeutic interventions, warranting further exploration in future research.

A possible direction for future research would be the integration of serum biomarkers into the study, aiming to correlate them with immunohistochemical findings. This could offer significant clinical advantages due to the ease of monitoring serum biomarkers in patient assessments.

## 5. Conclusions

This study offers valuable insights into the relationships between various clinical and histopathological factors in cutaneous melanoma. The strong correlations identified may improve the accuracy of both diagnosis and prognosis. A deeper understanding of these associations enables more precise tumour assessment and staging, which is essential for developing individualized treatment plans. The findings emphasize the value of incorporating multiple biomarkers, such as PRAME or p16, into routine clinical practice to better predict tumor behavior and guide therapeutic decisions. Ongoing research should continue to explore these relationships to support the development of targeted therapies that aim to improve patient outcomes. By applying these findings, clinicians can optimize patient management through more effective and personalized treatment approaches.

## Figures and Tables

**Table 1:** The minimum, maximum, and median percentage of positive cells for SOX10, Melan-A, HMB45, S100, PRAME, and p16.

	SOX10	Melan-A	HMB45	S100	PRAME	p16
Minimum	70%	0%	0%	30%	0%	0%
Maximum	100%	100%	100%	100%	100%	90%
Median	85%	90%	80%	90%	90%	50%

**Table 2:** The *p*-values for the Correlation Matrix.

	Age	Mitoses	Breslow Depth	Ki-67	SOX10	Melan-A	HMB45	S100	PRAME	p16
Age		0.01	0.091	0.012	0.859	0.043	0.002	0.380	0.803	0.022
Mitoses	0.010		<0.0001	<0.0001	0.524	<0.0001	<0.0001	0.020	0.007	0.090
Breslow Depth	0.091	<0.0001		<0.0001	0.486	<0.0001	0.002	0.031	0.002	0.032
Ki-67	0.012	<0.0001	<0.0001		0.437	0.001	0.004	0.125	0.001	0.044
SOX10	0.859	0.524	0.486	0.437		0.437	0.268	0.100	0.004	0.348
Melan-A	0.043	<0.0001	<0.0001	0.001	0.437		<0.0001	<0.0001	0.846	0.233
HMB45	0.002	<0.0001	0.002	0.004	0.268	<0.0001		0.001	0.695	0.182
S100	0.380	0.002	0.031	0.125	0.100	<0.0001	0.001		0.200	0.850
PRAME	0.803	0.007	0.002	0.001	0.004	0.846	0.695	0.200		0.883
p16	0.022	0.09	0.032	0.044	0.3348	0.233	0.182	0.850	0.883	

**Table 3:** Univariate logistic regression survival analysis.

Parameter	OR	95%CI	*p* Value
Age	1.037	0.99–1.08	0.0595
Breslow depth	1.175	1.06–1.33	0.0003
Mitoses	1.302	1.13–1.57	<0.0001
Ki-67	1.047	1.02–1.08	0.0006
SOX10	1.009	0.89–1.15	0.882
Melan-A	0.9646	0.93–0.99	0.0116
HMB45	0.9756	0.95–0.99	0.0153
S100	0.9602	0.91–1.01	0.0806
PRAME	1.059	1.001–1.21	0.0449
p16	0.9476	0.86–0.99	0.014

## Data Availability

All the data processed in this article are archived in the pathology department at the University Hospital of Bucharest, where the interventions were performed. The original data are available upon reasonable request.
